# Impact of Modern Communication in Transforming Dental Care

**DOI:** 10.3390/dj13100441

**Published:** 2025-09-25

**Authors:** Jasmine Cheuk Ying Ho, Hollis Haotian Chai, Michelle Zeping Huang, Edward Chin Man Lo, Chun Hung Chu

**Affiliations:** 1 Faculty of Dentistry, University of Hong Kong, Hong Kong, China; u3008515@connect.hku.hk (J.C.Y.H.); hchai89@hku.hk (H.H.C.); michellehuang@hsu.edu.hk (M.Z.H.); hrdplcm@hku.hk (E.C.M.L.); 2 Department of English, The Hang Seng University of Hong Kong, Hong Kong, China

**Keywords:** dentist-patient communication, digital communication, modern communication

## Abstract

**Background:** Effective dentist–patient communication is pivotal for quality dental care and patient satisfaction. Advances in technology, such as the application of digital dentistry, have modernized how dentists and patients interact. **Objective:** The objective of this study is to review traditional and modern communication methods and how the latter enhance patient engagement. **Methods:** A systematic search of PubMed, Scopus, and Web of Science (2000–2025) was conducted using keywords related to dental communication methods. Eligible studies underwent critical appraisal based on methodological clarity and relevance, and review quality was assessed using the SANRA framework. **Results:** While traditional methods remain foundational, digital advancements have significantly expanded communication channels. Teledentistry improves access through virtual consultations, electronic health records empower patients with transparency, and mobile apps facilitate convenient messaging and monitoring. Social media and interactive educational content enhance patient understanding and practice engagement. AI-driven tools further personalize patient interactions and automate administrative tasks. However, these modern strategies require careful implementation to ensure they meet clinical needs and adhere to strict privacy and security regulations. **Conclusions:** In conclusion, technological advancements have re-shaped dentist–patient communication, making it more flexible and efficient, thus enhancing patient engagement, satisfaction, and overall dental care quality. Future research should focus on conducting comparative and longitudinal studies to evaluate patient outcomes, satisfaction, and the long-term impact on the dentist–patient relationship across different hybrid communication models.

## 1. Introduction

Effective dentist–patient communication is foundational to clinical success, influencing treatment outcome, health literacy, and patient satisfaction [1,2,3]. Historically, dental interactions relied on traditional methods such as in-person consultations, printed educational materials, and phone calls. While these approaches fostered basic trust, they often struggled to address evolving patient expectations for convenience, accessibility, and personalized engagement. Geographic barriers, time constraints, and passive patient roles further limited the efficacy of conventional communication, contributing to care disparities and fragmented health outcomes [4,5]. The rapid digitization of healthcare, however, has created opportunities to reimagine these dynamics.

The shift to digital communication is driven by pressing needs: rising demand for telehealth post-pandemic [6], growing health literacy expectations [7], and the necessity to reduce no-show rates in overburdened practices [8,9]. Furthermore, patients are increasingly arriving at consultations having already researched their symptoms and treatment options online, shifting the dynamic of the traditional dentist–patient dialog and creating a need for clear communication to address both accurate and inaccurate information. Traditional models, which lack real-time interactivity and fail to empower patients as active decision-makers, are increasingly misaligned with contemporary lifestyles. For instance, marginalized populations face challenges accessing in-person care, while time-poor patients prioritize instant, on-demand interactions. Modern strategies—teledentistry, electronic health records, mobile apps, and social media—address these gaps by enabling virtual consultations, transparent treatment tracking, and proactive education. Teledentistry, for example, democratizes access for rural or mobility-limited patients, while electronic health records foster accountability through shared treatment plans. Interactive mobile apps reduce administrative friction, and social media builds community trust via educational content.

Despite their promise, the integration of these tools remains underexplored in dental literature. Existing studies often focus on isolated technologies rather than their collective impact on communication dynamics, patient autonomy, or long-term care quality. Additionally, ethical considerations—such as data privacy, digital equity, and the risk of depersonalization—require deeper analysis to balance innovation with patient safety. The objective of this study is to review traditional and modern communication methods and how the latter enhance patient engagement. By analyzing both opportunities and challenges, this work seeks to guide practitioners in adopting strategies that align with clinical needs, regulatory standards, and the evolving expectations of digitally empowered patients.

## 2. Materials and Methods

A systematic literature search was conducted using the electronic databases PubMed, Scopus, and Web of Science to identify relevant articles published between 2000 and 2025. The search utilized a combination of the following keywords: (‘dentist-patient communication’ OR ‘dental communication’) AND (‘face-to-face’ OR ‘in-person consultation’ OR ‘printed materials’ OR ‘telephone’ OR ‘digital communication’ OR ‘teledentistry’ OR ‘electronic health records’ OR ‘mobile apps’ OR ‘social media’ OR ‘artificial intelligence’). Studies were included if they were published in English in peer-reviewed journals, focused on communication methods in dental care. The retrieved articles were screened by title and abstract, and full texts of relevant studies were assessed for eligibility. Although no formal risk-of-bias instrument was applied because of the narrative design, we undertook a light critical appraisal during screening: studies were included when they reported primary data relevant to the review question and demonstrated adequate methodological clarity (appropriate design, interpretable outcomes, and sufficient sample description); opinion-only pieces, duplicates, and reports lacking primary outcomes were excluded. The Scale for the Assessment of Narrative Review Articles (SANRA) was applied to assess the quality of this review in order to improve the methodological transparency and scientific rigor of the literature search and review process (submitted as Supplementary File: File S1).

## 3. Traditional Communication Methods

Traditional communication methods include face-to-face interactions and printed materials. Traditional communication methods, such as face-to-face interactions remain crucial for conducting physical examinations, performing procedures, and offering personalized care. These methods of dentist–patient communication have long been the standard, which are deeply rooted in the history of dental practice.

### 3.1. Face-to-Face Interaction

Face-to-face interaction is the most fundamental and historically entrenched means of medical communication. It remains a vital means of communication in medical practice, forming the bedrock upon which trust, and rapport are built [10,11]. During in-person consultations, dentists have the unique opportunity to engage directly with patients, offering explanations of procedures, addressing questions, and alleviating concerns in real time.

The benefits of face-to-face interactions in medical care are substantial. They enable a level of personalized care that is difficult to replicate through other communication methods. A study on patients’ perspective on the mixing of face-to-face and remote consultation for palliative care revealed that patients consider face-to-face consultations facilitate communication [12]. Dentists can use visual aids, models, and diagrams to enhance their explanations, making complex dental procedures more comprehensible to patients [13,14]. This means also allows dentists to read non-verbal cues such as facial expressions and body language, which can provide valuable insights into a patient’s level of understanding, comfort, and anxiety. By interpreting these cues, dentists can adjust their communication style, ensuring that patients are fully informed and at ease. Moreover, patient trust is nurtured by empathetic and clear communication during in-person consultations [15,16,17]. This trust is vital for patient compliance, leading to better adherence to treatment plans, post-procedure care, and regular visits [18,19,20]. Direct engagement fosters a deeper understanding of patient needs, preferences, and concerns, allowing for more tailored and effective treatment plans. Additionally, the opportunity for immediate feedback and clarification helps to ensure that patients leave the consultation with a clear understanding of their dental health and the proposed treatment.

However, face-to-face interactions are not without their challenges. One significant limitation is the constraint of time. In busy dental practices, the demand to see a high volume of patients can lead to hurried consultations [21]. Dentists may feel pressured to prioritize efficiency over thoroughness, potentially compromising the quality of communication. Rushed interactions can result in incomplete information exchange, leaving patients with lingering questions or uncertainties about their treatment. This can adversely affect patient satisfaction and compliance, as well as the overall quality of care.

Additionally, an often-overlooked limitation of face-to-face consultations is their limited accessibility due to various factors. Geographic barriers, such as patients living in remote or rural areas, can prevent them from easily reaching healthcare facilities. Physical limitations, including mobility issues, disabilities, or health conditions, may also hinder patients from attending in-person appointments. Furthermore, time constraints related to busy schedules, work commitments, or transportation challenges can make it difficult for some patients to access face-to-face consultations consistently

Since face-to-face communication remains the cornerstone of high-quality dental care, to mitigate these time constraints, dental practices can implement structured communication protocols, such as the ‘teach-back’ method where patients repeat instructions in their own words or dedicate specific time slots for complex case discussions to ensure thorough patient education without compromising clinical efficiency [22].

### 3.2. Telephone Communication

Telephone Communication as one of the telehealth communication methods, has been a fundamental aspect of patient interaction for many years. It serves multiple essential functions within the medical practice, including scheduling appointments [23], answering inquiries, providing follow-up care [24], and addressing urgent concerns and triage assessment since the COVID-19 pandemic [25,26,27]. The convenience and immediacy of telephone communication make it an indispensable tool for maintaining continuous contact between dentists and their patients, particularly among older populations. However, this method also presents several challenges and limitations that can impact its effectiveness.

One of the primary advantages of telephone communication is its accessibility. Patients can easily contact their dental office from the comfort of their home or workplace without the need to visit the practice in person. Additionally, telephone communication is often used for quick inquiries, such as confirming appointment times, asking about office hours, or seeking advice on minor dental issues. Telephone communication also plays a critical role in providing follow-up care [24]. After a dental procedure, patients may have questions or experience symptoms that require clarification. A study found that brief telephone consultations can provide patients with reassurance and essential guidance [28]. These consultations help address immediate concerns and ensure patients correctly follow post-procedure instructions. This can be particularly important for procedures that have specific aftercare requirements, such as extractions, root canals, or surgeries.

Despite its advantages, telephone communication has notable limitations. One significant challenge is the lack of visual aids [29]. Dental care often involves complex procedures and detailed instructions that can be difficult to convey verbally. Without the ability to use diagrams, models, or visual demonstrations, patients may struggle to fully understand explanations or instructions provided over the phone. This can lead to miscommunication, confusion, and concerns of potential errors in following care guidelines [30]. Moreover, resolving issues over the phone can be time-consuming, especially when detailed discussions are required. Complex dental concerns often necessitate in-depth explanations and back-and-forth dialog, which can be cumbersome in a telephone conversation. This limitation is compounded by the fact that telephone communication lacks the personal touch of face-to-face interactions. Building rapport and trust with patients is more challenging without the benefit of non-verbal cues, which play a crucial role in effective communication.

Patients may also feel less comfortable asking questions or expressing concerns over the phone. The absence of a physical presence can make the interaction feel more impersonal, leading to incomplete information exchange. A study comparing face-to-face and telephone consultations in primary healthcare found that telephone consultations were generally shorter in duration than face-to-face meeting. Additionally, patients tended to disclose less information during telephone consultations, and doctors asked fewer questions compared to in-person interactions [31]. Patients might hesitate to voice their worries or seek clarification, resulting in gaps in their understanding and potentially affecting their compliance with treatment plans.

Language barriers and differences in communication styles can further complicate telephone interactions. Dental practices serve diverse patient populations, and not all patients may be fluent in the language used by the dental office staff. Misunderstandings due to language differences can hinder effective communication and impact the quality of care. Additionally, variations in communication styles, such as differences in tone, pacing, and formality, can lead to misinterpretations and discomfort during telephone conversations.

### 3.3. Printed Materials

Printed materials, such as brochures, pamphlets, informational leaflets, and 3D printed models have long been one of the staples in dentist–patient communication [13]. These materials are designed to inform patients about various aspects of their dental care, including treatment options, aftercare instructions, and general oral health tips. The use of printed materials in dental practices serves several important functions and offers a range of benefits that contribute to patient education and engagement [14]. 

One of the primary advantages of printed materials is their role as a tangible reference for patients [32]. Unlike verbal instructions given during face-to-face consultations, printed materials can be reviewed multiple times at the patient’s convenience. This is particularly useful for patients who may feel overwhelmed or anxious during their dental visit and may not fully absorb all the information provided by their dentist. Having access to a physical resource allows patients to revisit the information at their own pace, enhancing their understanding and retention of key details.

Printed materials also help patients recall and retain health information. A study examining the impact of printed materials on patient education in Chicago indicated that the use of printed take-home materials resulted in a notable increase in patients’ memory and retention of health-related details [33]. They serve as a reminder of important points, such as post-procedure care instructions or the steps involved in a particular treatment. This reinforcement is crucial for ensuring that patients follow through with their treatment plans and adhere to recommended oral hygiene practices. Furthermore, printed materials, such as illustrations and 3D-printed models can be customized to address concerns related to specific treatments or conditions. A study demonstrated that incorporating 3D-printed models into patient education significantly enhances patients’ understanding and reduces anxiety in Mohs micrographic surgery for skin cancer [34]. By providing detailed explanations and visual aids, they can help demystify specialized procedures and alleviate patient anxiety and build trust.

However, despite their benefits, printed materials have notable limitations. A significant drawback is that traditional printing practices in healthcare settings predominantly rely on paper, which carries substantial environmental consequences. These practices contribute to deforestation, higher energy consumption, greater waste production, and increased carbon emissions. In contrast, 3D-printed materials are argued to have a reduced environmental impact, such as reduced energy consumptions and carbon dioxide emissions, due to the use of additive manufacturing [35]. These advantages, however, depend on the printing technology and material used. It is suggested to minimize active print time per product, reduce printer idling time, select greener, biodegradable, or recyclable materials, and optimize printing orientation and geometry to prevent material waste [36]. Additionally, the static nature of printed materials makes them susceptible to becoming outdated quickly as new information or guidelines emerge. To ensure that the information remains accurate and relevant, dental practices must frequently update and reprint their materials. This process is not only time-consuming and costly but also poses environmental concerns.

## 4. Contemporary Communication Methods

The emergence of digital healthcare has introduced modern communication methods, enhanced patient–physician interactions and addressed evolving patient expectations in healthcare [37]. These modern techniques leverage technology to enhance accessibility, efficiency, and patient engagement. Telehealth services, for instance, have revolutionized the way consultations are conducted. Virtual consultations allow patients to discuss their concerns from the comfort of their homes, reducing the need for travel and making it easier for those with mobility issues or busy schedules to seek care. Through video calls, dentists can provide immediate feedback and guidance, which can be especially valuable for addressing urgent concerns or conducting follow-up consultations.

The integration of digital technologies into healthcare delivery offers significant opportunities for improving access, efficiency, and patient engagement. However, this shift also presents notable ethical and behavioral challenges, particularly regarding digital equity and the potential for depersonalization in patient–provider interactions and challenges of patients with manipulative tendencies. Addressing these issues is essential to fostering inclusive and compassionate dental care.

One primary concern pertains to digital equity, which encompasses disparities in availability of devices, internet access and technological proficiency. Limited internet connectivity and technological literacy can disproportionately affect vulnerable populations, including older adults and residents of rural areas. These groups often face structural barriers such as inadequate infrastructure, socioeconomic disadvantages, and lower digital literacy levels [38]. Consequently, reliance on digital communication may inadvertently exclude these populations from accessing timely and effective dental care, exacerbating existing health disparities.

Furthermore, the shift toward digital platforms can contribute to depersonalization in patient care. Reduced face-to-face interactions may weaken the development of trust and rapport—elements that are fundamental to effective dental care and patient satisfaction [1]. Trust is often built through non-verbal cues, empathetic gestures, and personal engagement, which can be diminished in virtual settings. This depersonalization may lead to decreased patient adherence, increased anxiety, and a sense of alienation, particularly among populations already vulnerable to healthcare disparities.

Patients exhibiting manipulative tendencies may selectively present symptoms or visual data in ways that are misleading, which can complicate accurate diagnosis and treatment. Similarly, individuals with dependency traits—often seen in certain patient groups—might overuse telehealth systems, unintentionally creating additional workload for practitioners and disrupting the clinical workflow.

### 4.1. Teledentistry

Telemedicine have revolutionized patient–physician communication, bringing a safe and a new level of convenience and accessibility to healthcare [39,40]. Teledentistry is recognized as a specialized branch within the broader domain of telemedicine and is defined as the provision of real-time and offline dental care such as diagnosis, treatment planning, consulting and follow-up care via digital communication technologies, such as video conferencing, mobile apps, and imaging software [41].

Teledentistry has emerged as a transformative solution, particularly for patients in remote areas or those facing mobility challenges. These virtual video consultations are a core component of teledentistry, proving particularly valuable for initial screenings, triage, and providing preliminary guidance. In rural or underserved regions, where access to dental care is often limited due to geographic barriers and a scarcity of dental professionals, teledentistry bridges this critical gap [42,43]. Studies have shown that teledentistry significantly improves access to preventive care [44,45,46,47] and early detection of oral diseases, including dental caries and oral cancer [42,48,49,50] in these populations, reducing the prevalence of untreated dental conditions and associated complications.

Teledentistry offers a valuable platform to enhance patient engagement by providing an additional, convenient channel for communication. This accessibility makes it easier for patients to seek advice, and follow up on their treatment plans without the need for frequent in-person visits. Furthermore, teledentistry promotes continuity of care by enabling dentists to monitor patients’ progress remotely and make timely adjustments to their treatment protocols as needed. Evidences show that patients are highly satisfied with teledentistry, particularly due to its convenience [51], seamless user experience, and dependable reliability [52].

However, teledentistry services come with certain limitations. Stable and reliable internet access, along with technological proficiency [53], are critical for seamless telehealth operations. The reliance on technology inherently creates a ‘digital divide,’ potentially excluding elderly, low-income, or rural populations without reliable internet or smart devices, thereby paradoxically exacerbating the very healthcare disparities teledentistry aims to solve [54]. Another limitation is that not all dental issues can be adequately assessed or treated through virtual consultations. Certain conditions, such as cavities, impacted teeth, or precise measurements for orthodontic work, require a physical examination and the use of specialized dental instruments. In such cases, teledentistry can serve as a supplementary tool rather than a complete replacement for in-person visits.

To aid clinicians in navigating these challenges and determining the appropriateness of remote care, Table 1 provides a practical triage rubric for patient suitability.

### 4.2. Electronic Health Records

Electronic health records have reformed dentist–patient communication, improving patient engagement and practice efficiency. By providing online access to treatment history, upcoming procedures, and educational materials, electronic health records empower patients to actively participate in their dental care [55,56]. Electronic health record refers to a comprehensive digital system that integrates a patient’s health information, medical history, and healthcare data across institutions and over time [57]. Electronic health records transform patients into active participants in their oral healthcare by enhancing their understanding of their health status and treatment plans. A key benefit of electronic health records lies in patient empowerment [58], facilitated by seamless access to personal health information. A UK-based study demonstrated that a significant majority of patients express a strong desire to engage more actively in their care through access to their health records [59]. Through secure online portals, patients can review their treatment history, track past and upcoming appointments, and access tailored educational resources related to their oral health conditions. This transparency not only fosters informed decision-making but also strengthens the patient–provider relationship, ultimately contributing to improved health outcomes.

Electronic health records also improve the quality of oral healthcare [60] and enhance coordination among dental providers [61], particularly when patients require care from multiple specialists, such as oral surgeons or periodontists. Electronic health records allow for the seamless sharing of patient records, ensuring continuity of care, minimizing redundant tests, and providing all providers with up-to-date information. Moreover, digital health records significantly enhance the efficiency of dental practice management. By automating administrative tasks—such as scheduling, billing, and record-keeping—electronic health records reduce the likelihood of errors and save valuable time for both staff and patients. 

Despite the numerous advantages of digital health records, their implementation and use present substantial risks. The full potential of the interoperability is often hindered by challenges in standardization across different Electronic health records systems and dental practices, which can create technical barriers to seamless information exchange. Another concern is the need for robust data security measures to protect patient privacy. A study exploring the concerns among nurses in the Mohs micrographic surgery regarding electronic health records highlighted privacy, confidentiality, security, and patient safety as their main issues [62]. To address these concerns, it is essential to ensure that sensitive information is securely managed and compliant with regulations. Dental practices, for instance, must invest in advanced technology solutions, such as encryption, secure access controls, and regular security audits, to safeguard patient data from breaches or unauthorized access. Additionally, educating patients about the importance of protecting their login credentials and recognizing phishing attempts can further enhance overall security.

### 4.3. Mobile Apps and Secure Messaging Systems

Mobile apps and secure messaging systems have become indispensable in modern dentistry, revolutionizing practice management and patient engagement by streamlining workflows and enhancing two-way communication between dentist and patient. These technologies facilitate radiographic image analysis, predictive modeling for risk assessment, virtual simulations to support treatment planning and secure messaging for practical post-treatment follow-up, allowing patients to ask minor questions and receive guidance without the need for a clinic visit [63].

One of the most significant advantages of mobile apps and secure messaging systems in healthcare is their ability to facilitate seamless and convenient communication between care providers and patients, fostering a direct dentist–patient relationship [64]. Features such as appointment scheduling, virtual consultations, and real-time messaging their dentist with pre- and post-treatment questions enable patients to engage with their care providers more efficiently and enhance their sense of involvement in their own care. This ongoing access transforms the patient role from a passive recipient of care to an active, engaged participant, fostering a sense of partnership and improving overall satisfaction. This also reduces the necessity for routine in-person visits, facilitating timely intervention and improving adherence to follow-up care protocols. Furthermore, mobile apps improve access to health information [65]. Despite variability in the quality of mobile apps as reported in the literature [66,67,68], they function as effective platforms for providing educational resources, health guidelines, and personalized care instructions. By empowering patients with knowledge about preventive care, treatment options, and post-procedural care, these tools promote better oral hygiene practices and reduce the incidence of dental diseases.

Beyond communication and education, mobile apps and messaging systems contribute to improved treatment adherences and decrease symptom severity. Research has shown that mobile apps are effective in monitoring symptoms and enhancing treatment adherence [69]. Features such as medication reminders, follow-up appointment notifications, and oral hygiene routines help patients stay consistent with their care plans. Furthermore, dental mobile apps have been shown to effectively reduce dental anxiety in children during non-invasive procedures [70]. These apps often incorporate interactive games, calming visuals, and step-by-step guides that help familiarize young patients with the dental environment, making the experience less intimidating and more engaging.

While the benefits of mobile apps and messaging systems are substantial, data security remains a critical concern in healthcare [71]. Dental practices must implement robust security measures to protect sensitive patient information and comply with regulatory standards. This includes using encrypted messaging systems, secure login protocols, and conducting regular security audits to safeguard patient data from breaches or unauthorized access [72]. Regular staff training and transparent communication with patients about data protection further build trust and ensure compliance with privacy regulations.

In addition, most mobile health apps and messaging systems are rooted in commercial underpinnings. The development of these tools typically requires substantial financial investment, which is often supplied by private companies whose primary motivation is financial gain rather than enhancing clinical outcomes. This commercial orientation influences the design and deployment of Health apps, primarily through monetization strategies such as subscription models or pervasive advertising. Such approaches pose challenges: subscription-based models may deter consistent use by patients and dental professionals, especially when the perceived clinical benefit is limited or unclear. Similarly, apps saturated with advertisements can undermine user trust, distract from core functionalities, and reduce perceived credibility. Furthermore, the effectiveness of these tools is not universal. Their uptake and utility are significantly influenced by patient age and technological literacy. It is important to maintain traditional options like phone calls to accommodate patients who may be less technologically proficient, such as older adults.

### 4.4. Educational Videos and Interactive Content

Educational videos and interactive content have emerged as powerful tools in modern dental practices, significantly enhancing patients’ understanding of oral health [73]. These contemporary communication methods enable dentists to explain procedures and oral health concepts in an engaging and accessible manner, catering to diverse learning styles. By serving as visual aids, educational videos can demystify dental procedures and simplify complex topics, fostering greater patient comprehension and reducing anxiety. For instance, a study demonstrated that showing a preoperative video on tooth extraction to patients effectively reduced fear and anxiety associated with dental extractions under local anesthesia [74].

Beyond videos, the adoption of digital imaging and 3D visualization software represents a significant advancement in patient education. Technologies such as intraoral scanners and treatment simulation software allow clinicians to visually demonstrate a patient’s current oral state and simulate potential outcomes of complex procedures. This ability to visually “show” possible results, rather than just “tell” them, demystifies treatments, manages patient expectations more effectively, and plays a crucial role in building trust and securing informed consent prior to initiating care.

Instructional videos on preventive care practices, such as proper brushing and flossing techniques, are highly effective in reinforcing healthy habits and promoting better oral hygiene. Additionally, studies have shown that animation is a highly effective educational tool for providing patient instructions, especially for younger patients [75,76]. By simplifying complex concepts and presenting them in an engaging format, animated videos make learning more accessible and enjoyable, fostering a positive attitude toward oral healthcare from an early age. Interactive content, including quizzes, tutorials, and interactive diagrams, further enhances patient education by actively involving patients in the learning process. These tools not only test knowledge but also reinforce key concepts, ensuring a deeper understanding and retention of oral health practices.

One of the primary challenges with using educational videos and interactive content is ensuring that the material is accurate, up-to-date, and relevant to the patient’s specific needs. Dental practices also have to invest time and resources in regularly updating their educational materials to reflect the latest advancements in dental care and address emerging health concerns.

### 4.5. Social Media and Online Reviews

Social media and online reviews have become indispensable tools for dental practices to connect with patients, share valuable information, and promote their practices [77,78]. A study revealed that a consideration number of patients are already comfortable engaging with their dentists through social media platforms like Facebook, Instagram, Twitter, and LinkedIn [79]. A primary educational function of these platforms is the dissemination of oral health tips, procedural videos, and awareness campaigns, allowing practices to reach and inform a broad audience.

One of the primary advantages of social media is its ability to effectively promote dental practices [80]. Dentists can share updates about their practice, announce new services, and provide insights into the latest advancements in dental care on these platforms. Beyond promotion, social media serves as a powerful tool for patient education. They can also use these platforms to share practical tips on oral hygiene, explain common dental procedures, and address frequently asked questions. By creating and sharing informative content—such as videos, infographics, and articles—dentists can demystify dental care, enhance patient understanding, and empower individuals to take proactive steps toward better oral health.

Online reviews and testimonials constitute a powerful yet double-edged sword for dental practices, playing a significant role in influencing patient decisions [80,81]. While positive reviews can greatly enhance a practice’s credibility and attractiveness, providing valuable social proof, their subjective and public nature also introduces significant reputational risks. Potential patients often turn to reviews to gauge quality, making their management a critical component of modern practice communication. This dual potential makes proactive and professional management of online feedback essential. Encouraging satisfied patients to leave reviews can help build a strong online reputation. Dentists can request feedback via email, during appointments, or through follow-up messages, ensuring it is convenient and accessible for patients to share their experiences.

However, social media and online reviews present several disadvantages that can hinder effective communication. One major concern is the widespread dissemination of misinformation on these platforms. Social media constitutes a chaotic mixture of unverified content, where patients often lack the specialized knowledge to distinguish evidence-based guidance from misleading claims. Systematic reviews have shown a substantial portion of dental and health-related content on social media is inaccurate or even harmful [82,83,84]. For example, quantitative analyses of popular health posts have found that a significant proportion contain misinformation, much of it potentially harmful to users [85]. This environment creates a fundamental challenge for dental professionals who must maintain evidence-based standards in public discourse. Unverified or inaccurate dental advice can quickly circulate, leading to patient confusion or inappropriate self-treatment [82,83]. This misinformation can erode trust, as individuals may prioritize unverified online sources over professional recommendations.

Beyond misinformation, social media promotes idealized and often unattainable aesthetic trends, such as the ‘California smile,’ which exert significant pressure on patient expectations. These trends may be medically unnecessary, technically unfeasible, or ethically questionable. This places the dental professional in a difficult position, potentially transforming their role from a healthcare practitioner grounded in evidence-based practice and bioethical standards to a service provider pressured to cater to commercially driven trends. Navigating patient demands influenced by these digital ideals without compromising professional integrity represents a critical modern ethical challenge.

Additionally, negative reviews or comments on social media can impact a practice’s reputation [81]. The subjective and anecdotal nature of these reviews presents significant limitations. They are often influenced by non-clinical factors such as practice ambiance or billing disputes, and may not accurately reflect the quality of clinical care. Furthermore, the threat of negative reviews can be misused as a form of coercion, pressuring clinicians into unjustified treatment plans. Given that platforms often make it difficult to remove malicious or dishonest reviews, dentists can be vulnerable to reputational damage based on misrepresentations rather than clinical reality. To mitigate the adverse effects of negative reviews, dental practices should address such feedback with constructive and empathetic responses, showcasing a dedication to resolving issues offline and upholding patient satisfaction.

### 4.6. Artificial Intelligence

The integration of Artificial Intelligence (AI) represents the most recent and transformative frontier in modernizing dentist–patient communication and practice management. Moving beyond static digital tools, AI leverages machine learning and natural language processing to create adaptive, predictive, and personalized communication pathways, fundamentally changing how patients interact with dental care systems [63].

A primary application of AI is through intelligent chatbots and virtual assistants. These systems are available 24/7 to handle routine inquiries, schedule appointments, answer frequently asked questions (FAQs), and provide basic oral health advice [86,87]. Beyond administrative functions, AI enables predictive analytics to revolutionize patient engagement and care outcomes. By analyzing data from electronic health records, patient communication histories, and even demographic information, AI algorithms can identify patients at high risk of missed appointments, poor treatment adherence, or the development of certain oral health conditions [88]. This allows dental teams to initiate proactive, targeted communication campaigns. AI also holds significant promise in enhancing clinical communication and patient education. AI-driven diagnostic tools can generate patient-friendly reports and visualizations, helping dentists explain complex conditions and the necessity of proposed treatments in a more transparent and understandable manner [89]. This augments the dentist’s expertise and empowers patients to make more informed decisions.

Despite their utility, AI-driven tools like chatbots are not without significant limitations. The adoption of AI in communication introduces new ethical and practical challenges that must be acknowledged. Data privacy and security concerns are paramount, as these systems process vast amounts of sensitive personal health information [71,72]. Ensuring algorithmic fairness and mitigating inherent biases (e.g., based on training data) is critical to prevent exacerbating healthcare disparities [90]. Furthermore, there is a risk of depersonalization and the erosion of the human touch that is central to therapeutic relationships. The empathy and nuanced understanding of a human dentist cannot be fully replicated by an algorithm [91]. Therefore, the most effective future model is likely a hybrid one, where AI handles efficiency-driven tasks and data analysis, freeing up the dental professional to focus on complex, empathetic, and high-value communication.

## 5. Conventional and Contemporary Communication Methods

To synthesize the array of communication methods discussed in Section 3 and Section 4, Table 2 presents a conceptual framework organized by primary function. This overview illustrates the complementary roles of traditional and contemporary tools within the broader ecosystem of dentist–patient interaction, providing a foundation for the subsequent comparative analysis of their specific advantages and limitations.

Table 3 summarizes the use and limitations of traditional and contemporary communication methods. The comparison between conventional and contemporary communication methods highlights the shift from a personal, in-person experience to a more flexible and efficient approach. While traditional methods emphasize personal interaction, contemporary methods prioritize convenience and accessibility. Modern communication strategies utilize dynamic, interactive formats that can be more engaging and empower patients with more information and options. However, the reliance on technology in contemporary methods contrasts with the physical presence required in conventional approaches.

The integration of technology in dental communication methods marks a significant shift towards a more patient-centered approach. By leveraging the strengths of both traditional and modern methods, dental practices can create a comprehensive communication strategy that meets the diverse needs of their patients. This hybrid approach ensures that patients receive the personal attention and reassurance they need while benefiting from the convenience and efficiency of modern technology. For example, digital platforms can be used for initial engagement and education, implementing secure messaging, email, or patient portals for providing appointment re-minders, pre-visit questionnaires, and educational materials about oral health. This can reduce in-office time, prepare patients beforehand, and encourage active participation. Additionally, teleconsultations can be used for routine follow-ups, oral health advice, or symptom assessment. This can enhance efficiency and patient preparedness. In-person consultations for physical examinations and complex procedures and trust-building. This allows dentists to discuss diagnosis, treatment options, and patient concerns. Printed materials can be used during in-person visits to enhance patients’ understanding of oral health.

## 6. Future Perspectives and Challenges

The evolution of dental communication is poised to continue at a rapid pace, shaped by emerging technologies and shifting patient expectations. Future advancements will likely be dominated by the deeper integration of Artificial Intelligence (AI) and machine learning for hyper-personalized patient engagement, predictive analytics, and automated practice management.

This review identified several areas requiring further research, including the long-term impact of digital communication on patient–dentist rapport, the development of best-practice guidelines for teledentistry, and strategies to address digital literacy and equity issues. Future research must also prioritize the development of robust, universally adopted cybersecurity standards for patient data and explore ethical frameworks to govern the use of AI in clinical communication. The future of dental communication lies in a hybrid model that thoughtfully integrates the empathy of traditional methods with the efficiency of modern technology. Ongoing training, research, and a patient-centered approach are essential to navigate this evolution successfully.

Crucially, this evolution mandates a parallel transformation in dental education and professional training. This begins with strengthening the existing curriculum for traditional communication skills like empathy, active listening, and patient education by employing proven methods such as role-playing, simulated patient interactions, and direct feedback [1]. The next generation of dental professionals must be equipped with dual competencies: mastering traditional interpersonal skills while achieving fluency in the effective, ethical, and secure application of digital communication platforms [92]. Therefore, dental curricula must integrate modules on digital literacy, teledentistry etiquette, and the ethical use of AI alongside foundational communication training. Embedding these digital competencies into the core curriculum is essential to prepare practitioners for a future where technology and patient-centric care are inextricably linked. Ultimately, the goal remains to leverage these tools to enhance, rather than replace, the human connection that is fundamental to quality dental care.

## 7. Conclusions

In conclusion, technological advancements have transformed dentist–patient communication, creating a flexible, efficient, and informative dialog. This evolution enhances patient engagement and satisfaction, ultimately improving the quality of dental care. It is crucial to emphasize that given the predominantly surgical and hands-on nature of dentistry, direct in-person clinician–patient interaction remains indispensable for comprehensive examination, diagnosis, and operative care. While both conventional and modern communication methods each offer unique advantages, integrating them can lead to a more comprehensive and effective approach. Digital and AI-enabled tools should be framed as powerful adjuncts that augment and support the core clinical encounter rather than replacements. However, the collective impact of integrated communication tools remains insufficiently explored within dental literature. Future studies should prioritize comparative and longitudinal investigations to better understand how various hybrid models influence patient outcomes, satisfaction, and the long-term dentist–patient relationship. Ultimately, the primary goal of effective dentist–patient communication is to ensure that patients are well-informed, comfortable, and actively engaged in their dental care journey.

## Figures and Tables

**Table 1:** Triage Rubric for Teledentistry Suitability and Decision Rules.

Criterion/Question	Yes/No	Action/Threshold
Are there red flags (e.g., new neurological deficits, severe pain, signs of systemic illness)?	Yes	In-person assessment mandatory. Do not manage remotely.
Is the presentation consistent with known, stable conditions?	Yes	Proceed with remote management, schedule follow-up.
Are symptoms inconsistent, exaggerated, or resistant to explanation?	Yes	Consider manipulative pattern; prioritize in-person evaluation and psychological assessment.
Is there evidence of recurrent overuse or dependence?	Yes	Evaluate for in-person assessment; consider multidisciplinary support.
Does the patient exhibit high anxiety, phobic behaviors, or significant distress?	Yes	Provide supportive communication; consider in-person evaluation or mental health referral.
Are communication barriers (e.g., language, cognitive impairment)?	Yes	In-person assessment recommended.

**Table 2:** A Functional Categorization of Dentist–Patient Communication Methods.

Category	Primary Function	Specific Methods (Section)
**In-Person Clinical** **Interaction**	Foundational clinical care, diagnosis, and trust-building	Face-to-Face Interaction (3.1)
**Remote Connection and Triage**	Providing access, remote consultation, and follow-up	Telephone Communication (3.2) Teledentistry (4.1)
**Information and** **Education**	Delivering educational content and enhancing patient understanding	Printed Materials (3.3) Educational Videos and Interactive Content (4.4)
**Practice Management and Coordination**	Streamlining administrative tasks and care coordination	Electronic Health Records (4.2) Mobile Apps and Secure Messaging Systems (4.3)
**Community Engagement and Reputation**	Building practice credibility and managing patient feedback	Social media and Online Reviews (4.5)
**Automated and Predictive Tools**	Automating tasks and enabling data-driven patient engagement	Artificial Intelligence (4.6)

**Table 3:** Comparative Analysis of Traditional and Contemporary Communication Methods in Dentistry.

Communication Methods	Advantages	Limitations	Ideal Use Context
**Traditional C** **ommunication Methods**			
**Face-to-Face Interaction**	Enables personalized care Builds trust and rapport	Requires considerable time	Complex diagnoses Manage anxious patients
**Telephone Communication**	Addresses urgent concerns Allows quick inquires and follow-up	Reduces the effectiveness of conveying complex procedures	Simple follow-ups Urgent issues Reach low digital literacy patients
**Printed Materials**	Enhance comfort of patients with multiple reviews	Raise concerns about the environment	Provide standardized instructions
**Contemporary Communication Methods**			
**Tele** **dentistry**	Facilitates virtual consultations Addresses disparities in oral healthcare access	Requires stable, reliable internet access, and technological proficiency	Initial screening Serve remote population
**Electronic** **Health** **Records**	Facilitate patient empowerment Enhance dentists’ coordination	Post concerns regarding patient privacy and confidentiality	Coordinate among dentists Maintain longitudinal treatment history
**Mobile Apps and Messaging**	Facilitate symptom monitoring Boosts treatment adherence	Post concerns regarding data security	Track post-operative symptoms Facilitate quick messages
**Educational Videos and Interactive Content**	Foster patient comprehension Reduce dental anxiety	Require resources and time to make the videos up to date	Demystify Professional Procedures Engage anxious patients
**Social Media and Online Reviews**	Aid in marketing dental practices Facilitate reputation building	Raise concerns about negative reviews and widespread dissemination of misinformation	Community engagement
**Artificial Intelligence**	Automates administrative tasks Enables predictive analytics	Raises data privacy concerns Potential for algorithmic bias	Handle high-volume inquiries provide basic support

## Supplementary Materials

https://www.mdpi.com/article/10.3390/dj13100441/s1, File S1: Scale for the Assessment of Narrative Review Articles—SANRA.
